# Resveratrol Reduces Prostate Cancer Growth and Metastasis by Inhibiting the Akt/MicroRNA-21 Pathway

**DOI:** 10.1371/journal.pone.0051655

**Published:** 2012-12-13

**Authors:** Sandeep Sheth, Sarvesh Jajoo, Tejbeer Kaur, Debashree Mukherjea, Kelly Sheehan, Leonard P. Rybak, Vickram Ramkumar

**Affiliations:** 1 Department of Pharmacology, Southern Illinois University School of Medicine, Springfield, Illinois, United States of America; 2 Department of Surgery, Southern Illinois University School of Medicine, Springfield, Illinois, United States of America; University of Kentucky College of Medicine, United States of America

## Abstract

The consumption of foods containing resveratrol produces significant health benefits. Resveratrol inhibits cancer by reducing cell proliferation and metastasis and by inducing apoptosis. These actions could be explained by its ability to inhibit (ERK-1/2), Akt and suppressing the levels of estrogen and insulin growth factor -1 (IGF-1) receptor. How these processes are manifested into the antitumor actions of resveratrol is not clear. Using microarray studies, we show that resveratrol reduced the expression of various prostate-tumor associated microRNAs (miRs) including *miR-21* in androgen-receptor negative and highly aggressive human prostate cancer cells, PC-3M-MM2. This effect of resveratrol was associated with reduced cell viability, migration and invasiveness. Additionally, resveratrol increased the expression of tumor suppressors, PDCD4 and maspin, which are negatively regulated by *miR-21*. Short interfering (si) RNA against PDCD4 attenuated resveratrol’s effect on prostate cancer cells, and similar effects were observed following over expression of *miR-21* with *pre-miR-21* oligonucleotides. PC-3M-MM2 cells also exhibited high levels of phospho-Akt (pAkt), which were reduced by both resveratrol and LY294002 (a PI3-kinase inhibitor). *MiR-21* expression in these cells appeared to be dependent on Akt, as LY294002 reduced the levels of *miR-21* along with a concurrent increase in PDCD4 expression. These *in vitro* findings were further corroborated in a severe combined immunodeficient (SCID) mouse xenograft model of prostate cancer. Oral administration of resveratrol not only inhibited the tumor growth but also decreased the incidence and number of metastatic lung lesions. These tumor- and metastatic-suppressive effects of resveratrol were associated with reduced *miR-21* and pAkt, and elevated PDCD4 levels. Similar anti-tumor effects of resveratrol were observed in DU145 and LNCaP prostate cancer cells which were associated with suppression of Akt and PDCD4, but independent of *miR-21*.These data suggest that resveratrol’s anti-tumor actions in prostate cancer could be explained, in part, through inhibition of Akt/*miR-21* signaling pathway.

## Introduction

A number of natural products, such as curcumin, isoflavone, resveratrol and epigallactocatechin-3-gallate (EGCG), show efficacy in controlling the growth and metastasis of various cancers [1]. Studies suggest that dietary intake of some of these products could aid in cancer prevention or enhance the efficacy of standard chemotherapeutic agents. Resveratrol (trans-3, 4′, 5-trihydroxystilbene) is a polyphenolic antioxidant found in peanuts, grapes and red wine [2], [3], which possesses significant health benefits [4]. This compound has shown beneficial effects in experimental cancer models, where it suppresses the initiation, promotion and progression of tumors [5]. Recent studies have implicated activation of the apoptotic pathway as a mechanism accounting for the anti-tumor benefits of resveratrol. For example, resveratrol inhibits cell proliferation and induces apoptosis of human prostate carcinoma DU145 cells [6] and acute lymphoblastic leukemia cells [7]. Resveratrol also produces cell cycle arrest of PC3 and DU145 androgen-insensitive cells [8]. In a Transgenic Adenocarcinoma Mouse Prostate (TRAMP) mouse model, resveratrol was shown to exert its anti-tumor action by increasing the expression of estrogen receptor-β and by decreasing insulin growth factor-1 (IGF-1) and extracellular signal regulated kinase 1/2 (ERK1/2) phosphorylation [9]. The latter actions of resveratrol could be produced by its ability to serve as an agonist/antagonist at the estrogen receptors on prostate cancer cells [10].

Preclinical studies indicate beneficial actions of resveratrol in preventing and treating cancer, with few associated side effects. A ten year epidemiologic study showed a greater than 50% reduction in breast cancer risk in women who ingested resveratrol by consuming grapes but not wine [11]. Several phase I and phase II clinical trials are currently underway for resveratrol at the National Institute of Health (http:/clinicaltrials.gov). For example, resveratrol is currently being investigated in Phase I trials against the Wnt pathway in colon cancer. Resveratrol is also being used in Phase II trials in lymphoma patients [12].

The efficacy of natural products, such as resveratrol, could be explained, at least in part, through their regulation of microRNAs (miRNAs) [13]. MiRNAs are small non-coding RNAs which regulate coding RNAs at the post-transcriptional level [14]. Several recent reports implicate miRNAs in the growth and metastasis of various cancers [15], [16]. Up-regulation of *miR-*21 has been detected in a number of cancers [14], including prostate cancer [17], [18], [19], suggesting that this miRNA could serve as a biomarker for these cancers. Several studies have implicated *miR-21* in oncogenesis. For example, *miR-21* regulates the growth of breast cancer cells (MCF7) *in vitro* and in a xenograft mouse model [20]. These investigators subsequently showed that *miR-21* regulated breast cancer metastasis by down regulating tumor suppressor genes, such as programmed cell death 4 (PDCD4) and maspin [21]. *MiR-21* has also been implicated in proliferation and metastasis of hepatocellular cancer cells by decreasing phosphatase and tensin homolog deleted on chromosome 10 (PTEN) [22]. In addition, *miR-21* regulates colon cancer intravasation and metastasis of colon cancer by targeting PDCD4 for down regulation [23]. Recent reports have also identified bone morphogenetic protein receptor II (BMPRII) [24] and lucine rich repeat (in FLII) interacting protein 1 (LRRFIP1) [25] as targets of *miR-21*. Inhibition of *miR-21* increased apopotosis of human glioblastoma cells [26], suggesting an anti-apoptotic role of this miRNA. The anti-apoptotic role of *miR-21* is also attributed to induction of the antiapoptotic Bcl-2 protein [20] and possibly to the activation of nuclear factor (NF)-κB [27].

In this study, we show that resveratrol reduces the viability and invasiveness of PC-3M-MM2 cells in culture and tumor growth in xenograft mouse model by inhibiting *miR-21* expression. This action is mediated by inhibition of Akt, an upstream regulator of *miR-21* gene.

**Figure 1:**
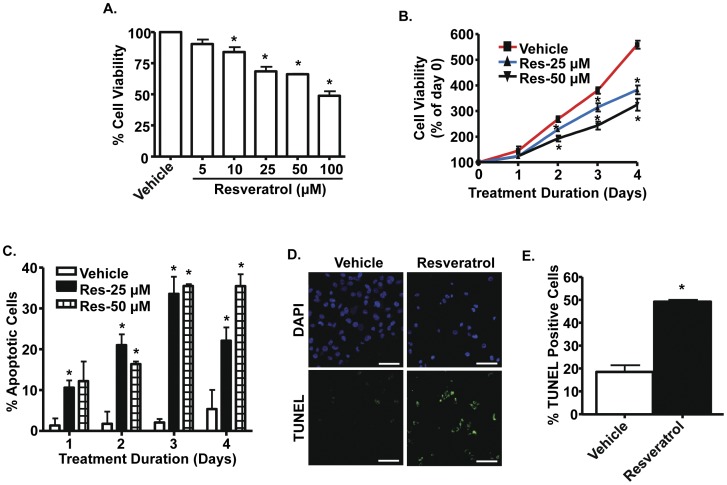
Resveratrol reduces PC-3M-MM2 cell viability by increasing apoptosis. *A,* MTS assay was performed on PC-3M-MM2 cells treated with vehicle or different concentrations of resveratrol (5–100 µM) for 72 h which showed that resveratrol dose-dependently reduced cell viability. ***B,*** Decrease in cell viability by resveratrol was time-dependent in the resveratrol-treated groups (25 and 50 µM) compared to vehicle. ***C,*** Resveratrol (25 and 50 µM) increased apoptosis of PC-3M-MM2 cells in a time-dependent manner, as determined by Annexin V-FITC and PI staining. ***D,*** Resveratrol (25 µM) increased apoptosis of PC-3M-MM2 cells, as determined by TUNEL assay. Cells were treated with resveratrol for 24 h and used for TUNEL assays, as described in Methods. DAPI staining was used to identify cell nuclei. Representative images are shown. Scale bar represents 100 µm. ***E,*** Bar graph indicate the percent of apoptotic cells in presence of 25 µM resveratrol shown in ***D***. Data indicate mean±SEM of at least 3 independent experiments. Asterisk (*) indicates statistically significant difference (p<0.05) from vehicle-treated cells.

## Materials and Methods

### Ethics Statement

All animal studies were conducted in accordance with the National Institutes of Health animal use guidelines and a protocol approved by the Southern Illinois University School of Medicine Laboratory Animal Care and Use Committee.

**Figure 2:**
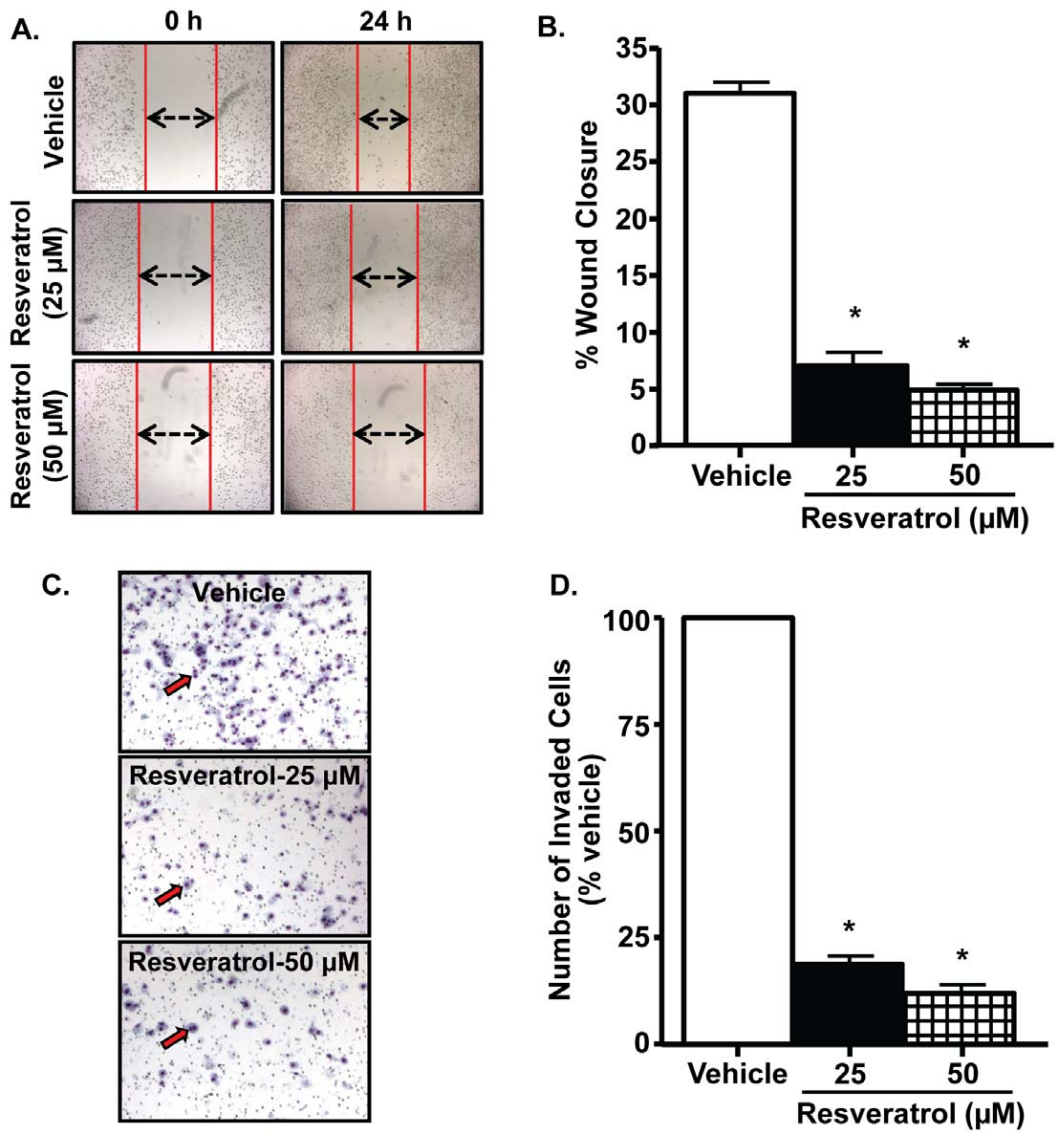
Resveratrol inhibits PC-3M-MM2 cell migration and invasiveness. *A, B,* Wound healing assays were performed on cells treated with vehicle or resveratrol (25 and 50 µM) for 24 h. Resveratrol significantly inhibited the migration of cells into the denuded areas, as indicated by double arrows ***(A)*** and in the bar diagram ***(B)***. ***C, D,*** Modified Boyden invasion chamber assay showed that resveratrol (25 and 50 µM) treatment for 24 h significantly inhibited PC-3M-MM2 cell invasion as indicated by red arrow ***(C)***. Representative images of the invasive cells per treatment group are shown. Data presented in the bar diagram ***(B, D)*** is mean±SEM of at least 3 independent experiments. Asterisk (*) indicates statistically significant difference (p<0.05) from vehicle-treated cells.

**Figure 3:**
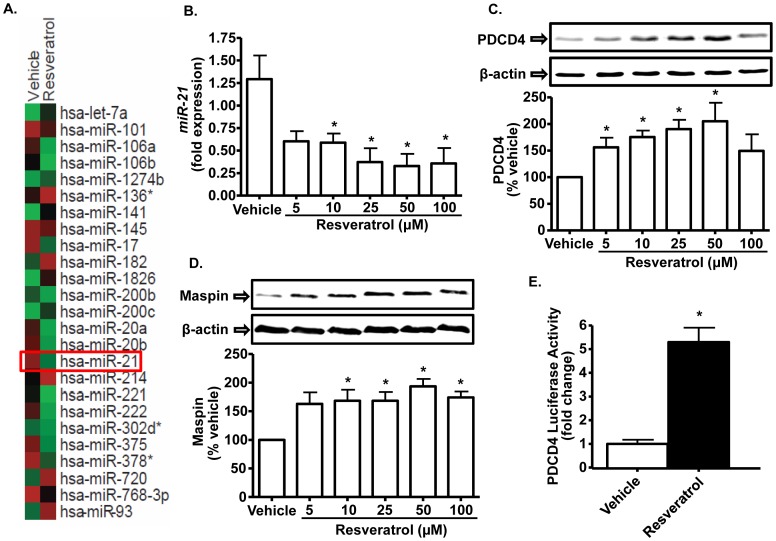
Resveratrol reduces *miR-21* expression and increases PDCD4 and maspin expression. *A,* MicroRNA gene array profile of genes regulated by resveratrol. PC-3M-MM2 cells were treated with vehicle or resveratrol (25 µM) for 24 h, after which total RNA was isolated. The samples were then subjected to microarray analysis for the detection of those *miRs* that are known to be regulated in prostate cancer [19] using Affymetrix gene chip (see Methods). Panels compare *miRNA* profile of vehicle and resveratrol-treated PC-3M-MM2 cells. The intensity of red and green color indicates the extent of up- and down-regulation of *miRNAs,* respectively. Similar studies were performed on three independent samples for each treatment group. The highest change was observed for *miR-21* as indicated. ***B,*** Resveratrol decreases *miR-21* levels in a dose-dependent manner. PC-3M-MM2 cells were treated with vehicle or resveratrol (5–100 µM) for 24 h, after which total RNA was isolated and *miR-21* levels were detected by quantitative RT-PCR using TaqMan® miRNA assay. ***C, D,*** Resveratrol increases the levels of PDCD4 and maspin in a dose-dependent manner. PC-3M-MM2 cells were treated with either vehicle or resveratrol (5–100 µM) for 24 h, after which whole cell lysates were prepared for Western blotting for the detection of PDCD4 and maspin, respectively. ***E,*** Resveratrol increased PDCD4 luciferase activity. Plasmid containing PDCD4 3′ UTR inserted downstream of luciferase coding sequence was transfected into the PC-3M-MM2 cells for 48 h. The cells were then treated with resveratrol (25 µM) for 24 h, after which cell lysates were prepared and subjected to luciferase assay. Data are presented as the mean±SEM of at least 3 independent experiments. Asterisk (*) indicates statistically significant difference (p<0.05) from vehicle-treated cells.

### Cell Culture

Highly invasive, androgen-independent human prostate carcinoma PC-3M-MM2 cells were obtained from Dr. Kounosuke Watabe (SIU School of Medicine, Springfield, IL). This cell line is derived from bone metastatic cell cultures of intra-cardiac injections of PC-3M-MM1 cells in nude mice [28]. PC-3M-MM1 cells in turn were derived from PC-3M cells [28], which are an aggressive variant of PC-3 cells [29]. Other prostate cancer cell lines, DU145 and LNCaP, were kindly provided by Dr. Daotai Nie (SIU School of Medicine, Springfield, IL). All cell lines were cultured in RPMI 1640 media (Gibco, Grand Island, NY) supplemented with 10% fetal bovine serum (Atlanta Biologicals, Lawrenceville, GA), 50 units/ml penicillin and 25 µg/ml streptomycin (Invitrogen, Carlsbad, CA). Cells were grown at 37°C in the presence of 5% CO_2_ and 95% ambient air. All the experiments were carried out in complete media using confluent monolayers, unless otherwise mentioned.

**Figure 4:**
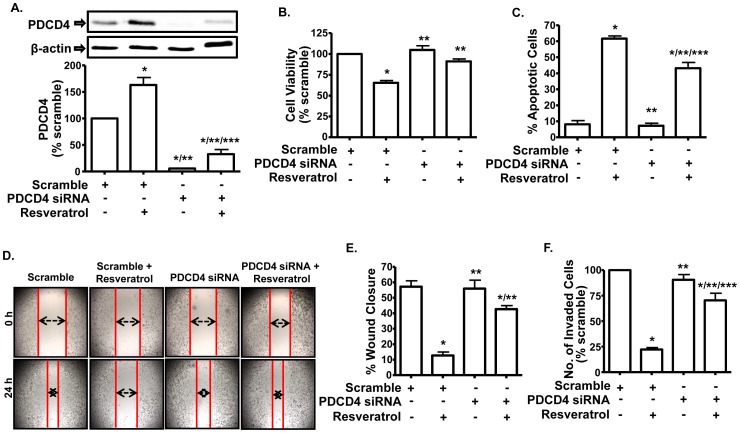
Down-regulation of PDCD4 attenuates the ability of resveratrol to decrease viability and invasiveness of PC-3M-MM2 cells. *A,* Western blot of the whole cell lysates of PC-3M-MM2 cells transfected with either scramble or PDCD4 siRNA (10 nM) for 48 h showed down-regulation of PDCD4 levels and reduced response to resveratrol (25 µM). Cells were exposed to resveratrol for 24 h after siRNA transfection. *B,* PDCD4 siRNA (10 nM) significantly reduced the ability of resveratrol (25 µM) to inhibit PC-3M-MM2 cell viability as determined by MTS assay. After 24 h of siRNA transfection, cells were seeded in 96 well plate and exposed to resveratrol for 72 h. *C,* PDCD4 siRNA partially reverses the ability of resveratrol to induce apoptosis in PC-3M-MM2 cells. Apoptosis assay was carried out by annexin V-FITC staining after 48 h of resveratrol (25 µM) treatment following 24 h of siRNA transfection. *D, E,* PDCD4 siRNA (10 nM) blocked the ability of resveratrol (25 µM) to inhibit migration of PC-3M-MM2 cells in wound healing assay as demonstrated by the double arrows *(D)*. Cells were transfected for 24 h, after which the wound was created and images were taken at time, 0 h. Cells were then treated with resveratrol for 24 h and images were taken again. Representative images are shown. The data from *(D)* are presented in bar diagram in *(E)*. *F,* PDCD4 siRNA (10 nM) reversed resveratrol-induced inhibition of PC-3M-MM2 cell invasion in modified Boyden invasion chamber assay. Cells were transfected in flasks for 24 h before seeding them in the top compartment. Data indicate mean±SEM of at least 3 independent experiments. (*), (**) and (***) indicate statistically significant difference (p<0.05) from scramble siRNA, from resveratrol+scramble siRNA and from PDCD4 siRNA treatment groups, respectively.

**Figure 5:**
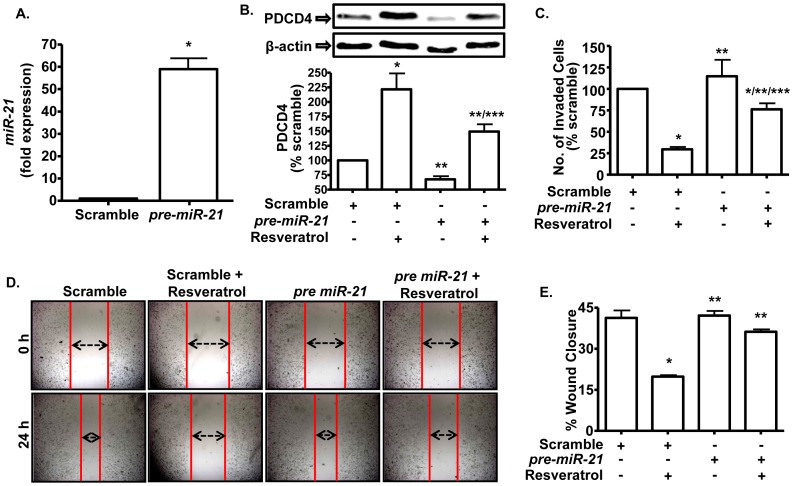
*Pre-miR-21* increases the levels of *miR-21* and attenuates the responses of resveratrol. *A,* Cells transfected with *pre-miR-21* (30 nM) showed increased levels of *miR-21*. Cells were transfected with *pre-miR-21* sequence for 48 h and were then used for determination of *miR-21* levels by quantitative RT-PCR using TaqMan® miRNA assay. Control cells were transfected with scrambled *pre-miR.* ***B,*** *Pre-miR-21* (30 nM) transfection decreased the levels of PDCD4 and attenuated the response of resveratrol (25 µM for 24 h), as determined by Western blotting. ***C,*** *Pre-miR-21* (30 nM) reversed resveratrol-induced inhibition of PC-3M-MM2 cell invasion in modified Boyden invasion chamber assay. ***D, E,*** Wound healing assay showing reversal of resveratrol-induced inhibition of PC-3M-MM2 cell migration by *pre-miR-21* (30 nM). Representative images are shown. Data in bar graphs are presented as mean±SEM of at least 3 independent experiments. Asterisks (*), (**) and (***) indicate statistically significant difference (p<0.05) from scramble-treated control, resveratrol+scramble *pre-miR* and *pre-miR-21* transfected PC-3M-MM2 cells, respectively.

### Reagents

Resveratrol and LY294002 were purchased from Sigma Chemical, Co (St. Louis, MO). *Pre-miR-21* oligonucleotide, *pre-miR* negative control, PDCD4 siRNA and negative control siRNA were purchased from Ambion (Houston, TX). Anti-PDCD4 antibody was from Epitomics, Inc (Burlingame, CA), whereas, antibodies against phospho-Akt was purchased from Cell Signaling Technology (Danvers, MA). Antibodies against maspin and Akt were purchased from Santa Cruz Biotechnology, Inc (Santa Cruz, CA) and anti-β-actin antibody was bought from Sigma Chemical, Co. All reagents and supplies were of highest available grade and were purchased from standard sources.

**Figure 6:**
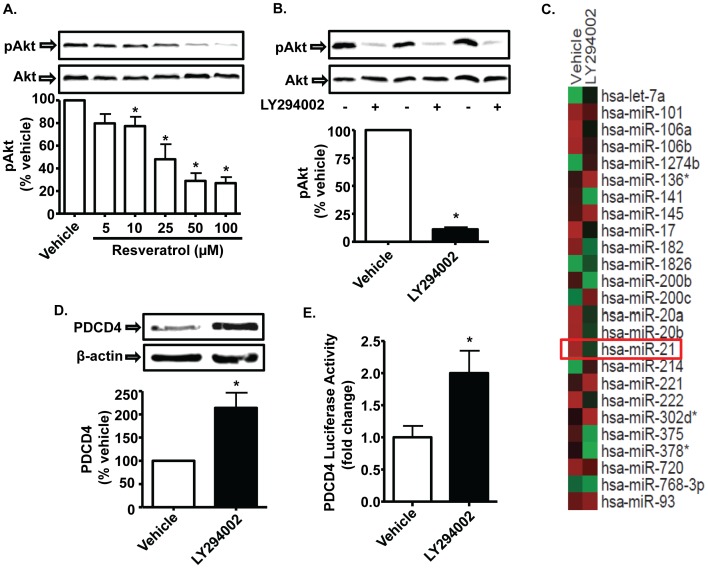
Resveratrol inhibition of Akt phosphorylation contributes to its regulation of *miR-21* expression and function. *A,* Resveratrol inhibits Akt phosphorylation in a dose-dependent manner. PC-3M-MM2 cells were exposed to vehicle or different doses of resveratrol (5-100 µM) for 24 h and whole cell lysates prepared from these cells were used for Western blotting. ***B,*** Inhibition of Akt phosphorylation by LY294002. PC-3M-MM2 cells were treated with LY294002 (10 µM), a PI3 kinase inhibitor, for 24 h and used for Western blotting to assess Akt activation. ***C,*** Akt inhibition reduced *miR-21* levels as determined by microRNA array analysis. PC-3M-MM2 cells were treated with vehicle or LY294002 (10 µM) for 24 h, after which total RNA was isolated. The RNA samples were then subjected to microarray analysis of *miRs*. The *miRs* which are known to be regulated in prostate cancer [19] are shown using Affymetrix gene chip. Panels compare *miRNA* profile of vehicle and LY294002-treated PC-3M-MM2 cells. The intensity of red and green color indicates the extent of up- and down-regulation of *miRNAs*, respectively. Similar studies were performed on three independent samples for each treatment groups. ***D,*** LY294002 increased PDCD4 levels. PC-3M-MM2 cells were exposed to LY294002 (10 µM) for 24 h and used for Western blotting. ***E,*** LY294002 increased PDCD4-luciferase activity. Plasmid containing PDCD4 3′-UTR downstream of the luciferase gene coding sequence was transfected into the PC-3M-MM2 cells for 48 h. The cells were then treated with LY294002 (10 µM) for 24 h, after which cell lysates were prepared and subjected to luciferase assay. Data in bar graph are presented as the mean±SEM of at least 3 independent experiments. Asterisk (*) indicate statistically significant difference (p<0.05) from vehicle-treated cells.

**Figure 7:**
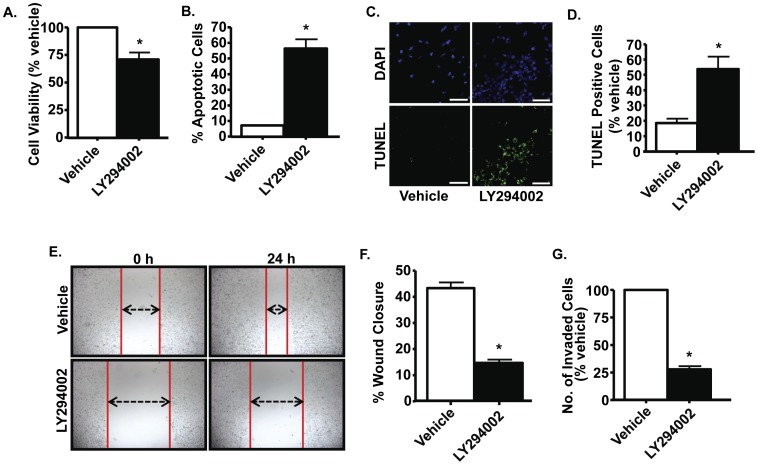
Activation of Akt is essential for survival, migration and invasion of PC-3M-MM2 cells. *A,* PC-3M-MM2 cells were treated with vehicle or LY294002 (10 µM) for 72 h and cell viability was determined by MTS assay. ***B,*** LY294002 (10 µM) treatment for 48 h induced apoptosis in PC-3M-MM2 cells as determined by Annexin V-FITC and PI staining which was quantified using flow cytometry. ***C,*** LY294002 (10 µM) increased apoptosis of PC-3M-MM2 cells, as determined by TUNEL assay. Cells were treated with LY294002 for 24 h and then used for TUNEL assay, as described in Methods. DAPI staining was used to identify cell nuclei. Representative images are shown. Scale bar is 100 µm. Bar graph ***(D)*** shows the percent of apoptotic cells in presence of 10 µM LY294002. ***E, F,*** LY294002 (10 µM) exposure for 24 h reduced migration of PC-3M-MM2 cells, as determined by wound healing assay. Representative images are shown. ***G,*** LY294002 (10 µM) exposure for 24 h reduced the invasiveness of PC-3M-MM2 cells, as determined by modified Boyden chamber assay. Data in bar graphs are presented as the mean±SEM of at least 3 independent experiments. Asterisk (*) indicates statistically significant difference (p<0.05) from vehicle-treated cells.

**Figure 8:**
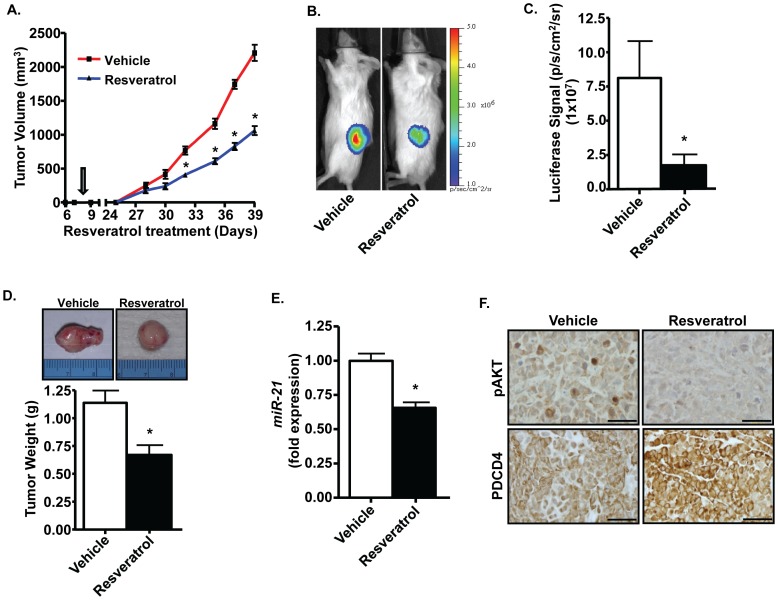
Resveratrol suppresses tumor growth *in vivo* by regulating *miR-21*. Resveratrol suppressed tumor growth *in vivo*. Twelve SCID mice were divided equally into two treatment groups- vehicle and resveratrol (20 mg/kg body weight). Drugs were administered by oral gavage every other day until the end of the study. Luciferase-tagged PC-3M-MM2 cells (1×10^6^) were injected into the flank region of each mouse on day 8 (arrow) of resveratrol treatment and then monitored for tumor growth. Resveratrol treated mice showed suppressed tumor growth ***(A)***, diminished luciferase signal ***(B, C)***, and smaller tumors ***(D)***. Representative images are shown in ***(B)*** and ***(D)***, while histograms in ***(C)*** and ***(D)*** represent mean±SEM of six mice from each treatment group. ***E,*** Tumors isolated from resveratrol-treated mice showed reduced levels of *miR-21* as determined by TaqMan® assay. ***F,*** Resveratrol regulates the expression of pAkt and PDCD4 in the tumor tissue. Isolated tumors from vehicle- and resveratrol-treated mice were sectioned for immunohistochemical staining for pAkt and PDCD4. Scale bar is 50 µm. Asterisk (*) indicates statistically significant difference (p<0.05) from vehicle-treated mice.

**Figure 9:**
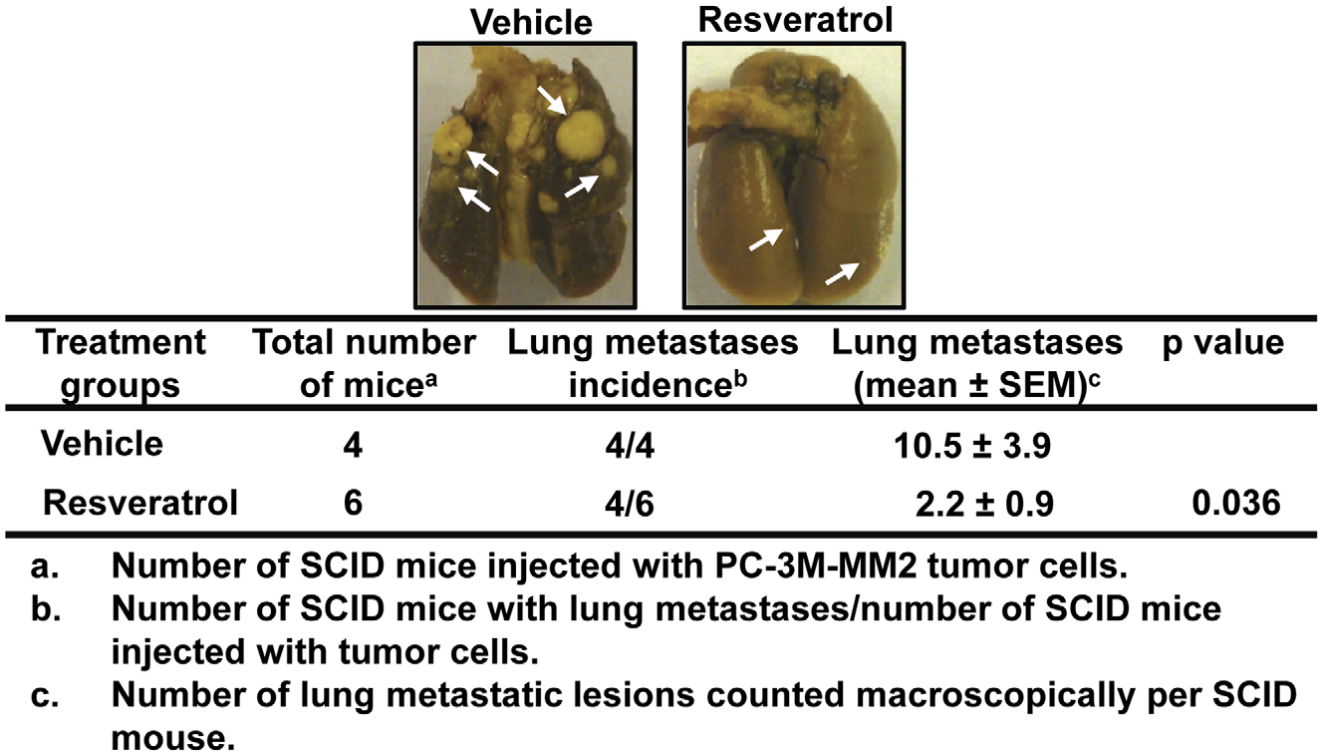
Resveratrol treatment reduced the incidence of lung metastasis in mice. SCID mice were treated with either vehicle (n = 4) or resveratrol (20 mg/kg body weight) (n = 6) through oral gavage, every other day until the end of the study, starting one week before injecting PC-3M-MM2 cells (1×10^6^), i.v. via tail vein. Mice treated with resveratrol showed significantly reduced number of metastatic lesions (arrow). Representative images of lungs from one mouse per treatment group are shown.

### Cell Viability Assay (MTS Assay)

*In vitro* PC-3M-MM2 cell proliferation was performed by using CellTiter 96® AQueous One Solution Cell Proliferation Assay Kit (Promega, Madison, WI), according to the manufacturer’s instructions. In brief, 2500 cells per well were seeded into a 96-well plate. After respective treatments, cells were allowed to grow for 72 h. After 72 h, 20 µl of CellTiter 96 AQueous One Solution reagent was added to each well in 100 µl of total volume of media. Cells were incubated for 3 to 4 h, and absorbance was recorded at 490 nm using an ELISA plate reader. The absorbance is directly proportional to the number of living cells and is expressed as a percent relative to vehicle-treated cells.

### Apoptosis Detection by Flow Cytometry

Cell death via apoptosis was determined by using FITC-AnnexinV Apoptosis detection kit (BD Pharmingen, San Diego, CA, USA) and quantified by flow cytometry as described previously [30]. Briefly, at the end of the treatment time**,** PC-3M-MM2 cells were washed once with phosphate buffered saline (PBS) and harvested in a 0.5% trypsin/EDTA solution at 37°C, centrifuged at 220×*g* for 5 min and then immediately re-suspended in the physiological buffer (1X) provided in the kit. Cells (1×10^5^ cells/500 µl) were then maintained in the dark for 15 min at room temperature with 5 µl of both propidium iodide and FITC conjugated annexin V, after which the samples were analyzed immediately by BD Biosciences *FACSCalibur* flow cytometer (San Jose, CA). The results were quantified using the CellQuest software (BD Biosciences, San Jose, CA).

### Apoptosis Detection by TUNEL Assay

Cell death via apoptosis in PC-3M-MM2 cells was detected by TUNEL assay using Fluorescein FragELTM DNA fragmentation detection kit (EMD Biosciences). Briefly, monolayers of PC-3M-MM2 cells were cultured on glass coverslips. At the end of treatment, cells were fixed with 4% paraformaldehyde. The cells were then *permeabilized* using proteinase K (1∶100 dilution) (provided in the kit) for 20 min at room temperature. At the end of incubation the cells were rinsed with 1X Tris-buffered saline (TBS). Following which they were incubated with 1X TdT equilibration buffer for 10–30 min. After the incubation was over, 60 µl of TdT labeling reaction mixture was applied on each coverslip and were placed in a humidified chamber and incubated for 1–1.5 h at 37°C. Next, the cells were rinsed twice with 1X TBS. Cells containing glass coverslips were mounted using Fluorescein-FragELTM mounting media. Excess of the mounting media was wiped off and the edges were sealed using nail polish. Slides were then imaged using an Olympus confocal microscope (Olympus America Inc., Melville, NY).

### Modified Wound-Healing Assay

Equal numbers of cells were plated in each well of twelve-well culture plates. After the cells reached 70% to 80% confluence, a line was scratched in the middle of the well using a pipette tip to create a wound. Differential interference contrast (DIC) images of the denuded area at three random fields per well were captured by a confocal microscopy just after the denudation (time, 0 h). Cells were then treated with different reagents and incubated for another 24 h, and images were again recorded. For quantitation, the area of the closing wound is measured from the denuded area and normalized to vehicle-treated controls. To study the effects of the PDCD4 siRNA and *pre-miR-21* on wound-healing of PC-3M-MM2 cells, the cells were transfected with PDCD4 siRNA, *pre-miR-21*and their respective negative controls for 24 h after which the wound was created and the images were taken at time, 0 h.

### Modified Boyden Invasion Chamber Assay

The ability of prostate cancer cells to migrate through matrigel-coated membranes was measured using 24-well BD Biocoat Matrigel invasion chambers (BD Biosciences, San Jose, CA). PC-3M-MM2 cells (5×10^4^) were suspended in the culture media without serum and were seeded on the top compartment of the invasion chamber followed by respective treatments. Complete media was added to the bottom chamber. At the end of 24 hours, the cell inserts were removed, and cells were carefully wiped from the top surface of the membrane with a cotton swab. The invasive cells adhering to the bottom surface of the membrane were stained with 100% methanol and 1% toluidine blue, respectively. The images were taken under a light microscope using a 20x objective. Total number of invaded cells was manually counted in four randomly chosen fields per treatment per insert. To study the effects of the PDCD4 siRNA and *pre-miR-21*, PC-3M-MM2 cells were transfected with PDCD4 siRNA, *pre-miR-21*and their respective negative controls for 24 h before seeding them on the top compartment.

### Western Blot Analysis

At the end of the treatment, PC-3M-MM2 cells were washed with ice cold 1X PBS and homogenized using a sonicator in ice-cold lyses buffer containing 50 mM Tris HCl, 10 mM MgCl2 and 1 mM EDTA in the presence of protease inhibitors mixture (Sigma) and phosphatase inhibitor 1 (1∶100) (Sigma). The whole-cell lysates were then used for western blotting as described previously [31]. Briefly, protein concentration was determined by Bradford method and equal amount of protein from each sample was mixed with solubilization buffer (20% SDS, 1M Tris, 20% glycerol and pinch of bromophenol blue) and heated on water bath at 95°C for 5 min. The samples were then resolved by SDS polyacrylamide gel electrophoresis. Proteins were transferred to nitrocellulose membranes, blocked in a solution containing 10X PBS, 10 mM EDTA, 20% of TritonX-100 and 5% low-fat skim milk for 1 h, and then incubated at 4°C overnight with the primary antibody. After three washes in blocking solution (blotto), blots were incubated with horseradish peroxidase-labeled species specific IgG secondary antibody for 1 h at room temperature, washed three times with 1X TBS containing 1% Tween20. This was followed by three washes with 1X TBS, without Tween 20. The blot was treated with ECL plus reagent (Pierce Biotechnology, Inc., Rockford, IL) and visualized using charged-coupled device LAS 4000 (Fujifilm North America Corporation, Valhalla, NY). Densitometric analysis of the bands was performed by using MultiGauge version 2.0 software. Individual bands were normalized to either Total-Akt or β-actin as the ratio of target protein. This was further normalized as % of control by taking the values of control as 100%.

### Oligonucleotide and Short Interfering (si) RNA Transfections

Synthetic *pre-miR-21* (30 ng) (ID # PM10206, Ambion, Austin, TX), PDCD4 siRNA (10 ng) (siRNA ID # s223740, Ambion) and their respective negative controls were delivered into PC-3M-MM2 cells using Lipofectamine™ RNAiMAX (Life Technologies Corp., Carlsbad, CA) as per manufacturer’s protocol. Briefly, PC-3M-MM2 cells were seeded in 6-well plates at 30% confluency. Next day, appropriate amount of *pre-miR-21* or PDCD4 siRNA and their negative controls were diluted in 100 µl serum-free medium and were incubated at room temperature for 15 to 20 minutes with 5 µl of Lipofectamine™ RNAiMAX transfection reagent to allow the formation of transfection complexes, which were added to the cell cultures by gently swirling the plates. The culture media was replaced with fresh media after 5–6 h and the cells were incubated for 48 h. For all other subsequent experiments, the same protocol was followed unless otherwise specified.

### RNA Isolation, Reverse Transcription and TaqMan Real-Time PCR

Isolation of total RNA from PC-3M-MM2 cells and tumor tissues was performed using QIAzol Lysis Reagent (Qiagen, Valencia, CA) as directed by the manufacturer. *MiR-21* cDNA was generated from 200 ng of total RNA which was reverse transcribed using *hsa-miR-21* qRT-PCR primer set (Applied Biosystems, Foster City, CA) and TaqMan® MicroRNA Reverse Transcription (RT) kit (Applied Biosystems). Each RT reaction contained 1X RT specific primer, 1X RT reaction buffer, 0.15 µl of 100 mM dNTPs, 50 U/µl MultiScribe RT enzyme and 3.8 U/µl RNase inhibitor. The 15 µl reaction mix were then incubated for 30 min at 16°C, 30 min at 42°C, and 5 min at 85°C and then held at 4°C in a PCR cycler. The real-time PCR was performed on Applied Biosystems StepOnePlus™ Real-Time PCR System using a standard TaqMan® PCR kit (Applied Biosystems) protocol. Briefly, following the RT step, 2 µl of the RT reaction was combined with 1 µl of 20X TaqMan® MicroRNA Assay (forward primer, reverse primer and probe) and 17 µl of TaqMan® Universal PCR Master Mix in 20 µl final volume. The reactions were incubated at 95°C for 10 min, followed by 40 cycles of 95°C for 15 s and 60°C for 1 min. Mature *miR-21* expression was calculated using the 2^-ddC^_t_ method relative to U6-snRNA and expressed as fold change. All TaqMan-PCRs were performed in triplicates.

### MicroArray Analysis

The miRNA microarray profiling was performed using Affymetrix GeneChip miRNA v1 arrays (Santa Clara, CA) according to manufacturer’s recommended protocol and was carried out by the CFG Microarray Core Facility (University at Albany, Rensselaer, NY). Briefly, 500 ng of the total RNA extracted from the cells was labeled by polyA polymerase addition using the Genisphere FlashTag HSR kit (Genisphere, Hatfield, PA). RNA was hybridized to the Affymetrix miRNA v1 array and scanned on a GeneChip Scanner 30007G as recommended by the vendor. The raw data was checked for quality using miRNA QC tool software (Affymetrix). Further analysis was done using GeneSpring Gx v11.3. The CEL files were imported and treated by the following workflow: background detection, RMA global background correlation, quantile normalization, median polish and log2-transformed. The entity list was then filtered to select for human small RNAs only. This list was further filtered to remove entities with low signal (bottom 20^th^ percentile) across all samples. The miRNAs previously shown to be differentially expressed in prostate cancer [19] were selected, subjected to the *t-test* for their statistical relevance and presented as tree view using Cluster 3.0 software.

### Luciferase Reporter Assay

Plasmids for the assay were gifted by Dr. Yin-Yuan Mo (SIU School of Medicine, Springfield, IL). 3′-untranslated mRNA sequence of PDCD4 having *miR-21* binding site was cloned downstream to the luciferase gene as described previously in Zhu S et al, 2008 [21]. PC-3M-MM2 cells were co-transfected with luciferase-tagged PDCD4 plasmid and pGL3 Renilla-luciferase using DNAfectin (Applied Biological Materials Inc., Richmond, BC, Canada) for 48 h. After the treatment time, luciferase activity was assessed by Dual-Luciferase Reporter Assay kit (Promega) according to the manufacturer’s protocol using a luminometer (Sirius, Berthold Detection System, Huntsville, AL). Renilla luciferase was used for normalization.

### In Vivo Studies

All In order to determine the *in vivo* anti-tumor effects of resveratrol, PC-3M-MM2 cells (1×10^6^) were subcutaneously injected into the flank region of 4-week old severely combined immunodeficient (SCID) male mice (Harlan Sprague-Dawley, Indianapolis, IN). Resveratrol (20 mg/kg body weight) suspended in PBS was administered to these mice via oral gavage every 2 days starting a week before tumor inoculation until the end of the study. The control group received vehicle (PBS) only. The subcutaneous primary tumor sizes were measured thrice a week with a caliper, and the tumor volume was calculated using the formula, tumor volume = (W^2^)×(L/2), where W represents the width and L represents the tumor length [32]. The tumor size was measured until the endpoint (tumor became as big as 2 cm long) after which the animals were sacrificed. Tumors were harvested and processed for real-time PCR and immunohistochemical studies. To determine the spontaneous metastatic ability, PC-3M-MM2 cells (1×10^6^) were injected i.v. via the tail-vein of SCID mice. Mice were administered either vehicle or resveratrol (20 mg/kg body weight) for 5 weeks, at the end of which all mice were sacrificed and number of macroscopic lung metastases were counted manually.

### In Vivo Imaging

In vivo imaging was performed to monitor the tumor growth in SCID mice. 100 µl of 15 mg/ml D-luciferin (RPI Research Products International Corp., Mt. Prospect, IL) was injected intraperitoneally 10 min before imaging. The animals were anesthetized using a gas anesthetic, isoflurane and bioluminescence images were captured using IVIS Imaging system (Xenogen, MA). Imaging and quantification of images were controlled by the acquisition and analysis software, Living Image.

### Immunohistochemistry (IHC) Analysis

Tumor samples which were harvested from mice were fixed in 4% paraformaldehyde and embedded in paraffin for sectioning. IHC staining was performed using 3, 3′-diaminobenzidine tetrahydrochloride (DAB) solution for the detection of PDCD4 and phospho-Akt. Briefly, tumor sections were deparaffinized, rehydrated ad immersed in 10mM sodium citrate buffer (pH 6.0) at 90°C water bath for 15 min for antigen retrieval. After cooling down to room temperature, the slides were dipped in 3% H_2_O_2_ for 15 min at room temperature to block endogenous peroxidase activity. The sections were then washed twice with PBS and blocked with 5% goat serum in PBS for 20 min at room temperature. The sections were incubated with each primary antibody for 12 h at 4°C after which they were incubated with horseshoe radish peroxidase (HRP) tagged secondary antibody (Vectastain ABC kit, Burlingame, CA) for 45 min at room temperature according to the manufacturer’s protocol. The peroxidase activity was identified by reaction with DAB solution kit (Vector Laboratories, Inc., Burlingame, CA) for 5 min at room temperature. Sections were then counterstained with hematoxylin and dehydrated with ethanol and then mounted in Cytoseal 60 (Richard-Allan Scientific, Kalamazoo, MI). Slides were imaged using an Olympus light microscope (Olympus imaging America Inc., Center Valley, PA) using Olympus DP controller software Ver. 2.3.1.231.

### Statistical Analysis

Values are expressed as mean±SEM of at least three independent experiments. Statistical significance was determined by Student’s *t* test or one way ANOVA with Tukey’s post-hoc test. P-values of <0.05 were considered statistically significant. Statistical procedures were carried out using PASW Statistics 18 (IBM SPSS, Chicago, IL) software.

## Results

### Resveratrol Inhibits PC-3M-MM2 Cell Viability

PC-3M-MM2 cells were cultured in vehicle or different concentrations of resveratrol (5–100 µM) for up to 4 days. Cell viability, determined by MTS assay, was dose-dependently reduced at 10, 25, 50 and 100 µM resveratrol (Fig. 1A). The onset of this reduction was day 2, using 25 and 50 µM resveratrol. Statistically significant reductions in cell viability were observed on days 2, 3 and 4 days following treatment with 25 and 50 µM resveratrol (Fig. 1B). Similarly, resveratrol (25 µM) inhibited the viability of LNCaP and DU145 cells, assessed over a 4 day period (Fig. S2 A and B).The decrease in cell viability by resveratrol could be explained, in part, to increased apoptosis of PC-3M-MM2 cells, as determined by Annexin V-FITC and propidium iodide (PI) staining using flow cytometry (Fig. 1C). Increases in cell apoptosis were observed throughout the treatment period, starting at 1 day and progressing to about 35% of apoptotic cells by days 3 and 4. The reductions in cell number were also associated with cell cycle arrest, as evidenced by decreased percentages of cells in G_0_/G_1_ phase and increased percentages in the S and G_2_/M phase by resveratrol (25 and 50 µM) (Fig. S1). Furthermore, resveratrol increased apoptosis of PC-3M-MM2 cells, as measured by TUNEL assay (Fig. 1D and E), from 19% to 49%. These data indicate that resveratrol is able to reduce cell viability by inducing apoptosis.

### Resveratrol Inhibits Invasiveness of PC-3M-MM2 Cells

The effect of resveratrol on the migration of PC-3M-MM2 cells was determined by “wound healing” assays. This assay measures the ability of cells to migrate into an area of a cell culture plate denuded of cells (wound). As shown in Fig. 2A and B, vehicle-treated cells produced ∼31% closures of the wound by 24 h. These responses were significantly attenuated by resveratrol at both 25 and 50 µM doses. Invasiveness of PC-3M-MM2 cells was also tested by modified Boyden invasion chamber assay which similarly revealed inhibition by resveratrol, assessed at 24 h (Fig. 2C and D). Experiments performed in LNCaP and DU145 cells showed similar responses to resveratrol. In these cells, resveratrol inhibited the wound closures from ∼22.5% to ∼11.5% and from ∼50% to∼31.9%, in LNCaP and DU145 cells, respectively (Fig. S2 C and D).These data support the conclusion that resveratrol inhibits the invasiveness of both androgen-dependent and –independent prostate cancer cells.

### Resveratrol Regulates *miR-21* Expression in PC-3M-MM2 Cells

Microarray was performed to examine miRNAs that were regulated by resveratrol, which could account for its growth inhibitory effects. Several miRNAs have been shown to be regulated by resveratrol in human SW480 colon cancer cells [33]. We investigated the expression of those miRNAs which were previously shown to be regulated in prostate cancer [19]. Out of the 25 miRNAs screened, 15 were down-regulated and 10 miRNAs were found to be up-regulated by resveratrol in PC-3M-MM2 cells (see Fig. 3A). Of interest, we observed a ∼1.9 fold reduction in the expression of the oncomir, *miR-21*. Previous studies have shown that resveratrol can regulate the expression of *miR-21* which contributes to its cardioprotective action [34]. We therefore examined the potential implications of down-regulation *miR-21* on the actions of resveratrol in subsequent studies.

Using TaqMan assay, we confirmed the results of down-regulation of *miR-21* by resveratrol from gene array studies. These assays showed that resveratrol dose-dependently reduced *miR-21* expression, with ∼35% and ∼55% inhibition observed at 5 and 100 µM resveratrol, respectively (Fig. 3B). The resveratrol concentration associated with ∼50% inhibition (IC_50_) was 25 µM. However, experiments performed in LNCaP and DU145 cells did not show inhibition of *miR-21* expression following treatment with resveratrol (data not shown). The inhibition of *miR-21* expression was associated with dose-dependent increases in PDCD4 and maspin, two targets of *miR-21* (Fig. 3C and D). The effective concentration producing 50% response (EC_50_) also averaged 5–10 µM resveratrol. These data confirm the finding that *miR-21* gene is a potential target of resveratrol in PC-3M-MM2 cells. Peak stimulations of PDCD4 and maspin (∼200%) were obtained with 50 µM resveratrol, with no further increases observed at the 100 µM concentration (Fig. 3C and D). Increases in PDCD4 were associated with elevations in PDCD4 luciferase activity (Fig. 3E), suggesting a functional role of resveratrol mediated via *miR-21*. As in PC-3M-MM2 cells, studies performed in LNCaP and DU145 cells similarly demonstrated that resveratrol significantly increased the levels of PDCD4 (Fig. S3 B and F), suggesting a common response of resveratrol in these different cell lines.

Targeting PDCD4 by siRNA resulted in ∼94% knockdown in the levels of this protein and attenuated its absolute induction observed with resveratrol (Fig. 4A). Furthermore, PDCD4 siRNA reversed the anti-proliferative action of resveratrol. In PC-3M-MM2 cells treated with scrambled siRNA, resveratrol reduced cell viability by ∼35%. However, in cells pretreated with PDCD4 siRNA, the response of resveratrol was significantly attenuated (Fig. 4B). PDCD4 siRNA also attenuated resveratrol’s response with apoptotic cell death in these cells (Fig. 4C). In addition, while resveratrol reduced the invasiveness of PC-3M-MM2 cells treated with scrambled siRNA, this response was significantly reduced in cultures treated with PDCD4 siRNA, as indicated by both wound healing (Fig. 4D and E) and invasion chamber assays (Fig. 4F).

Further evidence supporting *miR-21* as a target of resveratrol was obtained in PC-3M-MM2 cells transfected with *pre-miR-21* in order to over-express this *miR*. *Pre-miR-21* is processed by an RNAse III enzyme, Dicer, to a 22 base pair double stranded RNA with a two nucleotide 3′ overhangs. One strand of this RNA duplex forms the mature miRNA that is then incorporated into RNA silencing complex (RISC), which allows it to functionally suppress expression and translation of its targeted RNAs [14], [35]. Effective transfection was evidenced by increased *miR-21* levels in the transfected cells compared to cells transfected with a scrambled control (Fig. 5A). The levels of PDCD4 were significantly reduced, as anticipated from previous studies showing that *miR-21* binds to the 3′ UTR region of the *PDCD4* mRNA and enhances its degradation [20], [21]. These results suggest that *pre-miR-21* is effectively processed in PC-3M-MM2 cells to functionally over-express *miR-21*. Over-expression of *pre-miR-21* renders these cells partly resistant to the PDCD4 stimulatory action of resveratrol (Fig. 5B). However, resveratrol significantly increased PDCD4 levels in these cells, attesting to its ability to suppress the high basal expression of *miR-21*. Cells over-expressing *miR-21* were also more resistant to the action of resveratrol to suppress invasiveness, as indicated in wound healing and invasion chamber assay (Fig. 5C–E). Overall, these data support *miR-21* as an essential target of resveratrol for mediating the growth and invasiveness of PC-3M-MM2 cells *in vitro*.

### Inhibition of *miR-21* Expression by Resveratrol Involves Akt

Previous studies have proposed that Akt is a direct regulator of *miR-21* expression [36]. We therefore examined whether this signaling protein is a target of resveratrol. PC-3M-MM2 cells exhibit high pAkt levels, which were inhibited by resveratrol in a dose-dependent manner. Approximately ∼52% inhibition was evident at 25 µM resveratrol and ∼73% inhibition observed at 100 µM resveratrol (Fig. 6A). The response of 100 µM resveratrol, which appeared to represent a maximum for this compound, was less than LY294002, a known inhibitor of Akt activity (Fig. 6B). Like resveratrol, LY294002 inhibited *miR-21* expression (Fig. 6C) and increased the levels of PDCD4 (Fig. 6D) and PDCD4 luciferase activity (Fig. 6E). However, LY294002 did not produce any significant change in the levels of maspin (data not shown). These results support the conclusion that inhibition of Akt by resveratrol could contribute to its suppression of *miR-21* expression. While the induction of PDCD4 is consistent with a decrease in *miR-21* levels, it is unclear why the levels of maspin were not regulated. Additional experiments were performed in LNCaP and DU145 cells to determine whether resveratrol inhibits pAKT levels in these cells. Resveratrol significantly inhibited pAkt levels in these prostate cancer cell lines without affecting the levels of Akt ( A and E). Similar responses were observed with LY294002 (Fig. S3 C and G). Overall, these studies suggest that inhibition of Akt phosphorylation could serve as a reasonable mechanism for reducing *miR-21* levels and increasing the expression of PDCD4.

Additional experiments were performed to test whether the functional responses of resveratrol could be explained by inhibition of the Akt pathway. LY294002 reduced the viability and increased the apoptosis of PC-3M-MM2 cells, as determined from MTS assay (Fig. 7A), and Annexin V-FITC and PI staining (Fig. 7B) and TUNEL assay (Fig. 7C and D), respectively. In addition, LY294002 inhibited PC-3M-MM2 cell invasiveness. Wound closures in vehicle-treated cells averaged ∼43%, while they averaged ∼15% in cultures treated with LY294002 (Fig. 7E and F). Similarly, LY294002 significantly reduced cell invasion through matrigel (Fig. 7G). Taken together, these data suggest that Akt plays an essential role in mediating the viability and invasiveness of PC-3M-MM2 cells and could represent a viable target for inhibition by resveratrol.

### Inhibition of Prostate Cancer Growth and Metastasis by Resveratrol is Associated with Decreased *miR-21* Expression

In order to determine the *in vivo* efficacy of resveratrol against prostate cancer, we tested this drug in xenograft model of prostate cancer in SCID mice. These mice were administered vehicle or resveratrol by oral gavage every other day starting 1 week prior to subcutaneous injections of PC-3M-MM2 tumor cells into their flanks. Palpable tumors were detected by treatment day 28 and tumor volumes were measured over an additional 11 days in both groups. Tumor growth increased exponentially in the vehicle-treated mice, but the growth in resveratrol-treated mice was significantly reduced beyond day 30. Mean tumor volumes were reduced by ∼50% in the resveratrol-treated group (Fig. 8A). Tagging of the PC-3M-MM2 cells with the luciferase gene allowed for whole animal imaging in a Xenogen imager, upon administration of luciferin. Representative images taken at the end of the treatment period are shown in Fig. 8B. The analyzed data indicate substantial reductions in bioluminescence in the animals treated with resveratrol, compared to vehicle-treated controls (Fig. 8C). In addition, the weights of the excised tumors were significantly reduced by resveratrol (Fig. 8D). Analysis of tumor samples showed that the expression of *miR-21* was significantly reduced by resveratrol (Fig. 8E). Furthermore, these tumors showed lower levels of Akt and elevated expression of PDCD4 (Fig. 8F). These data support the efficacy of resveratrol on tumor growth.

Additional studies were performed to determine the ability of resveratrol to inhibit the incidence of lung metastasis of prostate cancer cells in SCID mice. These mice were treated with either vehicle or resveratrol every other day starting 1 week prior to injecting PC-3M-MM2 cells i.v. via tail vein until the end of the study. After 5-weeks animals were sacrificed, the lungs were removed and macroscopic metastatic lesions were counted manually. Resveratrol significantly inhibited the incidence of lung metastases in these mice as compared to the vehicle-treated mice (Fig. 9). Four of four mice treated with vehicle showed lung metastasis, while four of six mice treated with resveratrol showed metastatic lesions in the lungs. In addition, the number of metastatic lesions were 10.5±3.9 in vehicle-treated mice administered PC-3M-MM2 cells and 2.2±0.9 in mice treated with resveratrol. Taken together, this data suggest that oral administration of resveratrol not only inhibit prostate cancer tumor growth but also inhibit metastasis in SCID mice. Overall, these data support the contention that inhibition of the Akt/*miR21* axis could contribute the antitumor efficacy of resveratrol in prostate cancer.

## Discussion

The current data demonstrate that Akt/*miR-21* axis is an important target of resveratrol for mediating survival and invasiveness of PC-3M-MM2 prostate cancer cells. These actions are produced, at least in part, through the phosphorylation of Akt and/or the induction of *miR-21* targeted genes, such as PDCD4. As such, we show that over-expression of *miR-21* or inhibition of PDCD4, antagonizes the anti-tumor actions of resveratrol. Overall, this study highlights that Akt/miR-21 pathway is mediating the anti-tumor actions of resveratrol in prostate cancer.

*MiR-21* is an oncomir which plays an important role in regulating various cellular processes to enhance cancer cell growth and invasiveness. The expression of *miR-21* is high in androgen-independent prostate cancer cell lines (such as PC3 and DU145) and low in LNCaP cells which are androgen-dependent prostate cancer cells [37]. It is proposed that the androgen/androgen receptor complex binds to the promoter region of *miR-21* and induces its expression [38]. Interestingly, the resulting high expression of *miR-21* is believed to promote androgen resistance, presumably by regulating a number of genes. *MiR-21* regulated genes include myristoylated alanine-rich protein kinase c substrate (MARCKS), PDCD4, maspin and tropomyosin-1 [14], [39]. *MiR-21* negatively regulates the levels of MARCKS, which is believed to control cell motility by interacting with actin cytoskeleton [39], [40]. As such, cells treated with antisense *miR-r-21* exhibited increased MARCKS levels and reduced invasiveness. Down-regulation of MARCKS by siRNA was able to increase the invasiveness of DU-145 prostate cancer cells [37]. Several mechanisms could account for the anti-tumor response of PDCD4. This protein suppresses protein translation by inhibiting eukaryotic initiation factor 4A activity [41]. PDCD4 also inhibits trans-activation of the activator protein (AP)-1 promoter by c-Jun [42] and thereby inhibits its growth promoting functions.

Several pieces of evidence support the conclusion that resveratrol targets *miR-21* for inhibition in PC-3M-MM2 cells. For example, resveratrol inhibits *miR-21* expression in PC-3M-MM2 cells and increase the protein levels of key targets, such as PDCD4 and maspin. Knockdown of PDCD4 reduced the ability of resveratrol to mediate the growth and invasiveness of PC-3M-MM2 prostate cancer cells. Furthermore, resveratrol inhibited the expression of the *miR-21* target, PDCD4, in PC-3M-MM2-derived tumors from a SCID-mouse tumor model. Overall, these data provide evidence that *miR-21* is a viable target of resveratrol for mediating its antitumor actions. However it should be noted that resveratrol regulates the expression of other miRs, including miR-20a and MiR-20b which could also contribute to its overall antitumor action.

Our data support the contention that Akt plays a major role in the regulation of *miR-21* expression in PC-3M-MM2 prostate cancer cells. The expression of the phosphorylated Akt (active form) is high in naive PC-3M-MM2 cells and is suppressed by resveratrol. We show that inhibition of pAkt by LY294002 decreased *miR-21* expression and increased PDCD4 levels. LY294002 mimicked resveratrol in its ability to decrease cell invasiveness. Thus, inhibition of Akt activation represents an important mechanism underlying the reductions in the growth, invasiveness and survival of prostate cancer cell lines by resveratrol. Recent studies have shown that Akt could directly phosphorylate PDCD4 at Ser^67^ and Ser^457^ leading to decreased transactivation of activator protein-1 (AP-1) promoter by c-Jun [43]. The Akt pathway was also shown to mediate serum-induced proteasomal degradation of PDCD4 in ovarian cancer [44]. It is possible that the direct Akt pathway is essential for regulating PDCD4 levels in DU145 and LNCaP cells, since in these cells resveratrol was unable to inhibit *miR-21* expression. Accordingly, different pathways for regulating PDCD4 expression may be active in different prostate cancer cell lines. Regardless of these differences, regulation of PDCD4 serves as the common mechanism underlying the anti-tumor actions of resveratrol in these cells.

What mechanism(s) regulate the high levels of pAkt in PC-3M-MM2 cells is not known. One possibility is that the high levels of growth factors in the serum in which these cells are cultured. IGF-1 blood levels are positively associated with increased risk of advanced stage prostate cancer [45]. Previous studies have also documented that IGF-1 regulates the growth of normal prostate. For example, IGF-1 deficient mice exhibit small prostate sizes [46], while administration of IGF-1 promotes prostate growth [47]. Inhibition of IGF-1 receptors attenuated the proliferation of prostate cancer cells in culture [48]. The levels of IGF-1 is high in androgen-independent prostate cancer [46], suggesting that this growth factor might serve as an alternate to androgens in mediating Akt activation and high *miR-21* expression in this type of prostate cancer.

Another potential regulator of *miR-21* expression includes TGF-β. TGF-β has been shown to increase *miR-21* expression by enhancing processing of *pri-miR-21* to *pre-miR* by Drosha [49]. Several proteins recruited to this task include SMAD 1/5, SMAD 2/3 and RNA helicase (p68) [49]. High levels of TGF-β are found in prostate cancers [50], suggesting that they could serve as a positive regulator of *miR-21* expression. Activation of the ERK1/2 MAP kinase pathway can also promote *miR-21* expression, through activation of AP-1 transcription factor. The *miR-21* promoter possesses AP-1 binding sites which could serve as targets for activated AP-1 complex produced through ERK1/2 activation [51]. It has also been shown that the transcription factor, signal tranducer and activator of transcription 3 (STAT3), could also induce *miR-21* expression in memory T cells obtained from patients with Sezary syndrome [52]. Exposure of these cells to IL-21 resulted in activation of STAT3 and induction of *miR-21*. *MiR-21* has also been shown to regulate inflammation [53].

Recent studies have implicated *miR-21* in the development of cancer resistance. For example, increased expression of *miR-21* is associated with development of resistance of glioblastoma multiforme cells to the chemotherapeutic agent, tenoposide, by downregulating a nuclear factor (NF)-κB inhibiting protein [25]. Constitutive activation of NF-κB is linked to chemotherapeutic resistance [54], [55]. In addition, inhibition of NF-κB has been shown to sensitize tumors to chemotherapeutic agents [56]. Overall, these studies support an integral role of *miR-21*/NF-κB interaction in the development of chemotherapeutic resistance in cancers. A recent study also indicated that interferon increases the expression of *miR-21* and reduces apoptosis of prostate cancer cells through activation of STAT3 [57]. These studies link *miR-21* to the pathogenesis of prostate cancer and suggest that targeting this factor or its downstream effectors could represent effective therapeutic approaches in the treatment of both androgen-sensitive and hormone refractory prostate cancer.

In summary, we have identified *miR-21* and Akt as novel targets of resveratrol for mediating inhibition of prostate cancer growth, survival and invasiveness. Our data support the hypothesis that resveratrol uncouples a putative growth factor-mediated activation of Akt, leading to the induction of *miR-21* expression or reductions in the levels of PDCD4. These factors control the growth and invasiveness of prostate cancer cell.

## Supporting Information

Figure S1 **Cell cycle analysis of exponentially growing PC-3M-MM2 cells treated with resveratrol.** PC-3M-MM2 cells (1×10^5^/well) were seeded in a 12 well plate in RPMI 1640 media. Cells were then either treated with vehicle or 25 and 50 µM resveratrol for 72 h following which the cells were collected by trypsinization and centrifugation. Cell pellets was washed once with ice cold PBS and then suspended in 0.1% Triton X-100 in PBS containing 100 µg/ml RNase A and 50 µg/ml propidium iodide, and incubated for 15 min on ice. Fluorescent emission was quantified by BD Biosciences *FACSCalibur* flow cytometer (San Jose, CA). The table shows the percentage of cells in G_0_/G_1_, S, and G_2_/M phase under each treatment condition. The figures are representative of at least 3 independent experiments. Asterisk (*) indicates statistically significant difference (p<0.05) from vehicle-treated cells.
(TIF)

Figure S2 **Resveratrol reduces cell viability and cell migration in LNCaP and DU145 cells.** ***A, B,*** 25 µM resveratrol reduced cell viability of LNCaP cells ***(A)*** and DU145 cells ***(B)*** in a time-dependent manner as determined by MTS assay. ***C, D,*** Wound healing assays were performed on LNCaP ***(C)*** and DU145 ***(D)*** cells that treated with either vehicle or resveratrol (25 µM) for 24 h. Resveratrol significantly inhibited the migration of cells into the denuded areas, as indicated by double arrows and in the bar diagram. Data in bar graphs are presented as the mean±SEM of at least 3 independent experiments. Asterisk (*) indicates statistically significant difference (p<0.05) from vehicle-treated cells.
(TIF)

Figure S3 **Up-regulation of PDCD4 by resveratrol is mediated via inhibition of Akt in both LNCap and DU145 cells.** ***A–D,*** Inhibition of Akt and up-regulation of PDCD4 by resveratrol and LY294002 in LNCaP cells. To check the inhibition of Akt phosphorylation by resveratrol LNCaP cells were treated with either vehicle or resveratrol (25 µM) for 6 h and whole cell lysates prepared from these cells were used for Western blotting ***(A)***. Similarly, LNCaP cells were treated with either vehicle, resveratrol (25 µM) or LY294002 (10 µM) for 24 h, after which whole cell lysates were prepared for Western blotting for the detection of PDCD4 and Akt ***(B, C, D)***. ***E–H,*** Inhibition of Akt and up-regulation of PDCD4 by resveratrol and LY294002 in DU145 cells. DU145 cells were treated with either vehicle or resveratrol (25 µM) for 6 h and whole cell lysates prepared from these cells were used for Western blotting to check the inhibition of Akt phosphorylation by resveratrol ***(E)***. Similarly, DU145 cells were treated with either vehicle, resveratrol (25 µM) or LY294002 (10 µM) for 24 h, after which whole cell lysates were prepared for the detection of PDCD4 and Akt by Western blot ***(F, G, H)***. Data in bar graphs are presented as the mean±SEM of at least 3 independent experiments. Asterisk (*) indicates statistically significant difference (p<0.05) from vehicle-treated cells.
(TIF)
