# Supplementary figures and images for: Resveratrol Reduces Prostate Cancer Growth and Metastasis by Inhibiting the Akt/MicroRNA-21 Pathway
Source: PLoS One. 2012 Dec 13;7(12):e51655. doi: 10.1371/journal.pone.0051655 (PMC3521661; doi:10.1371/journal.pone.0051655)

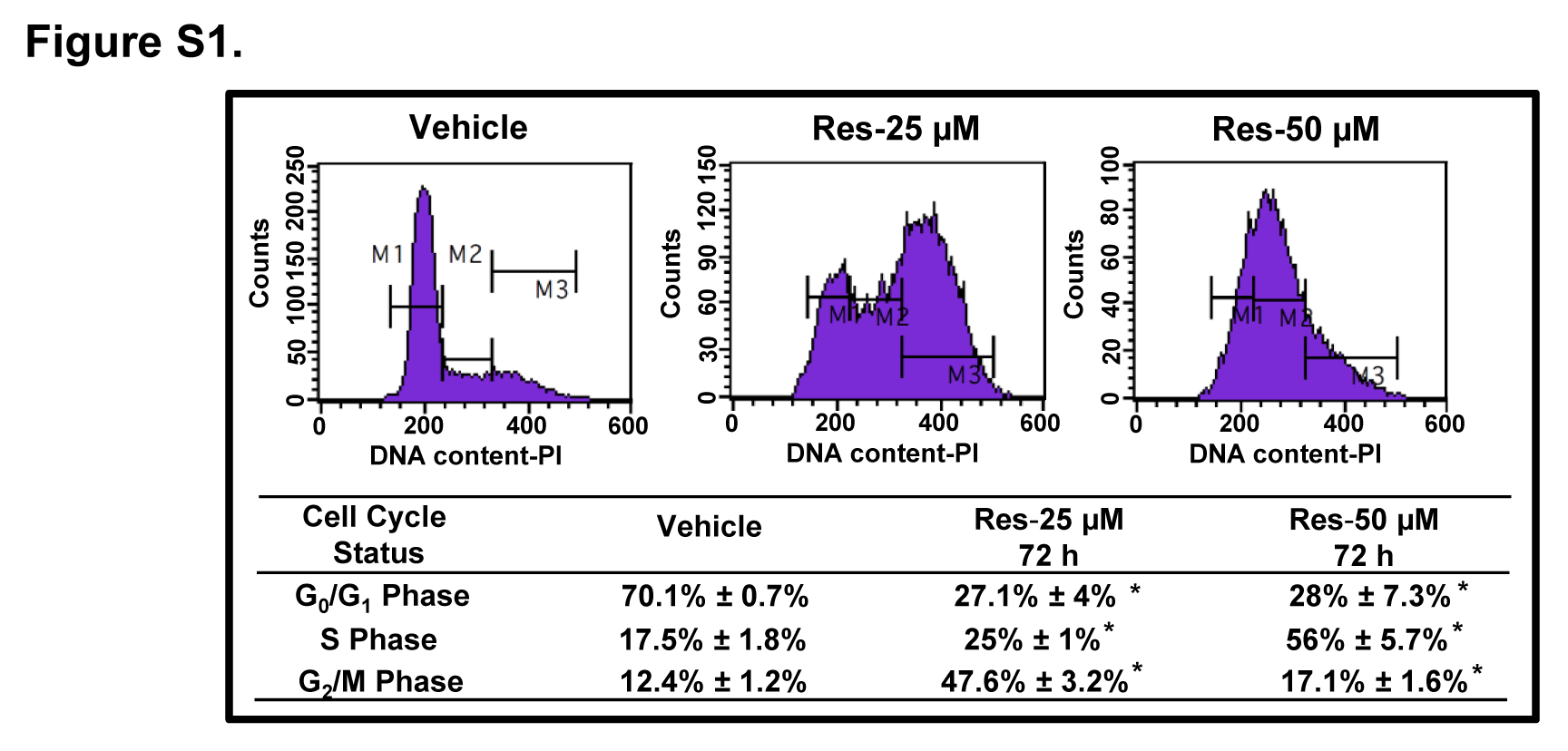

Supplement: Figure S1 — Cell cycle analysis of exponentially growing PC-3M-MM2 cells treated with resveratrol. PC-3M-MM2 cells (1×10^5^/well) were seeded in a 12 well plate in RPMI 1640 media. Cells were then either treated with vehicle or 25 and 50 µM resveratrol for 72 h following which the cells were collected by trypsinization and centrifugation. Cell pellets was washed once with ice cold PBS and then suspended in 0.1% Triton X-100 in PBS containing 100 µg/ml RNase A and 50 µg/ml propidium iodide, and incubated for 15 min on ice. Fluorescent emission was quantified by BD Biosciences FACSCalibur flow cytometer (San Jose, CA). The table shows the percentage of cells in G0/G1, S, and G2/M phase under each treatment condition. The figures are representative of at least 3 independent experiments. Asterisk (*) indicates statistically significant difference (p<0.05) from vehicle-treated cells. (TIF) [file pone.0051655.s001.tif]

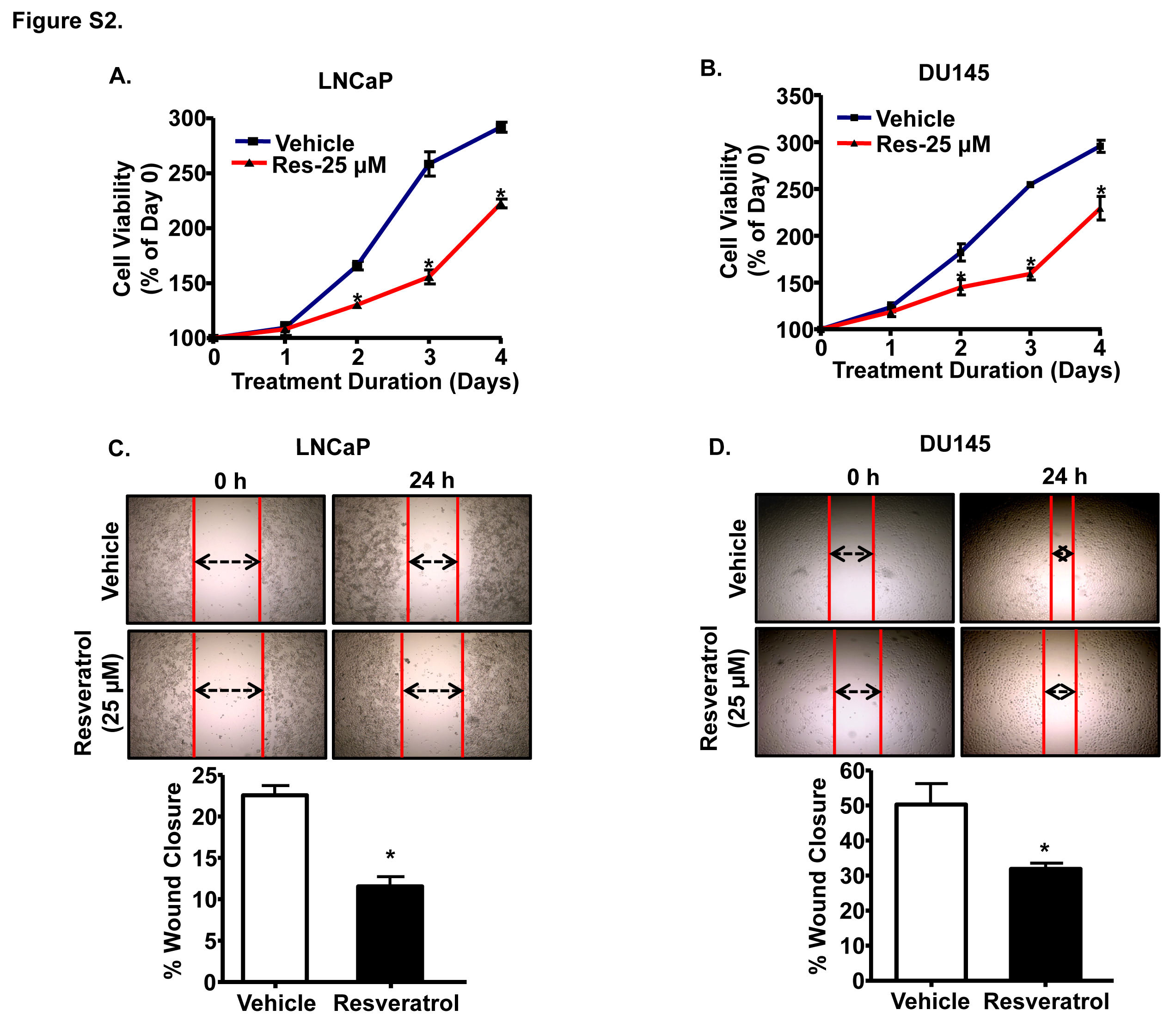

Supplement: Figure S2 — Resveratrol reduces cell viability and cell migration in LNCaP and DU145 cells. A, B, 25 µM resveratrol reduced cell viability of LNCaP cells (A) and DU145 cells (B) in a time-dependent manner as determined by MTS assay. C, D, Wound healing assays were performed on LNCaP (C) and DU145 (D) cells that treated with either vehicle or resveratrol (25 µM) for 24 h. Resveratrol significantly inhibited the migration of cells into the denuded areas, as indicated by double arrows and in the bar diagram. Data in bar graphs are presented as the mean±SEM of at least 3 independent experiments. Asterisk (*) indicates statistically significant difference (p<0.05) from vehicle-treated cells. (TIF) [file pone.0051655.s002.tif]

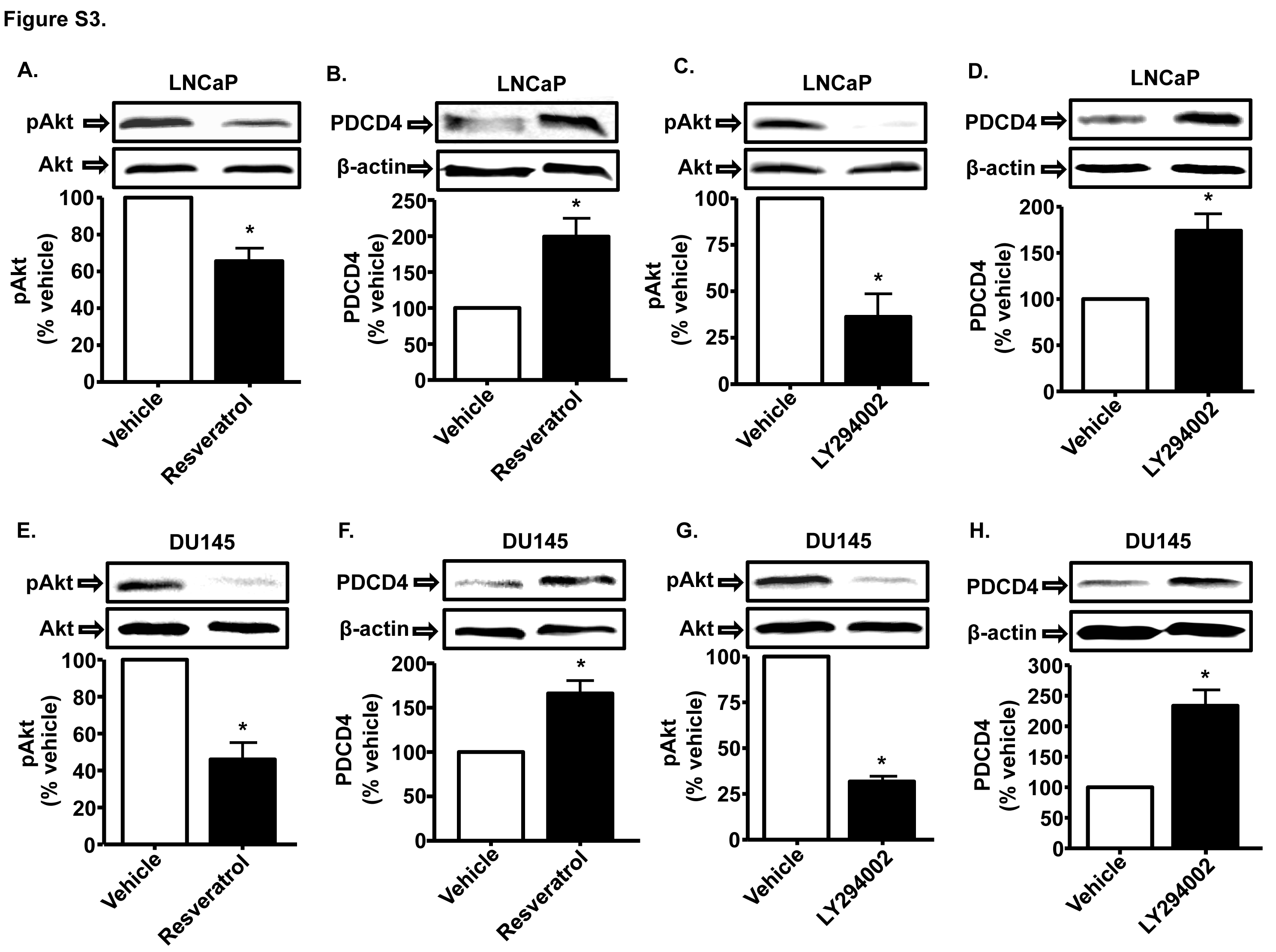

Supplement: Figure S3 — Up-regulation of PDCD4 by resveratrol is mediated via inhibition of Akt in both LNCap and DU145 cells. A–D, Inhibition of Akt and up-regulation of PDCD4 by resveratrol and LY294002 in LNCaP cells. To check the inhibition of Akt phosphorylation by resveratrol LNCaP cells were treated with either vehicle or resveratrol (25 µM) for 6 h and whole cell lysates prepared from these cells were used for Western blotting (A). Similarly, LNCaP cells were treated with either vehicle, resveratrol (25 µM) or LY294002 (10 µM) for 24 h, after which whole cell lysates were prepared for Western blotting for the detection of PDCD4 and Akt (B, C, D). E–H, Inhibition of Akt and up-regulation of PDCD4 by resveratrol and LY294002 in DU145 cells. DU145 cells were treated with either vehicle or resveratrol (25 µM) for 6 h and whole cell lysates prepared from these cells were used for Western blotting to check the inhibition of Akt phosphorylation by resveratrol (E). Similarly, DU145 cells were treated with either vehicle, resveratrol (25 µM) or LY294002 (10 µM) for 24 h, after which whole cell lysates were prepared for the detection of PDCD4 and Akt by Western blot (F, G, H). Data in bar graphs are presented as the mean±SEM of at least 3 independent experiments. Asterisk (*) indicates statistically significant difference (p<0.05) from vehicle-treated cells. (TIF) [file pone.0051655.s003.tif]
